# Commentary: Oxygen regulation of breathing through an olfactory receptor activated by lactate

**DOI:** 10.3389/fnins.2016.00213

**Published:** 2016-05-10

**Authors:** Mariarosaria Santillo, Simona Damiano

**Affiliations:** Dipartimento di Medicina Clinica e Chirurgia, Università di Napoli “Federico II”Naples, Italy

**Keywords:** carotid body, lactate, *Olfr78*, oxygen sensing, NOX, blood pressure

Carotid body (CB) is the main systemic arterial blood oxygen sensor assuring the homeostatic response to changes in oxygen tissue availability. In the CB, type I cells respond to acute or chronic hypoxic stimuli with increased neurotransmitter release and subsequent increased discharge of the post-synaptic carotid sinus nerve afferents reaching the brainstem cardiorespiratory centers (Kumar and Prabhakar, 2012).

The mechanism leading to neurotransmitter release by type I cells is an increase of extracellular Ca^++^ influx through Ca_v_ channels that follows membrane depolarization induced by a decrease of K^+^ permeability. This mechanism is shared by the other stimuli to which, in addition to hypoxia, CB responds, i.e., hypercapnia or acidosis. Nevertheless, not yet well-defined are the transduction mechanisms whereby hypoxia signals to K^+^ channels. The current scenario provides for the existence of multiple oxygen sensors acting simultaneously to ensure adequate cardiorespiratory response to hypoxia. In several chemosensory preparations was shown the direct inhibition of specific domains of oxygen sensing K^+^ channels (KO_2_) by hypoxia (Lopez-Barneo et al., 2001). However, according to different hypothesis, O_2_ sensing is not an intrinsic characteristic of K^+^ channel, rather K^+^ channel closure is a secondary phenomenon that follows the effect of hypoxia on other mitochondrial or membrane oxygen sensing molecular targets (Kumar and Prabhakar, 2012).

Chang et al. (2015) in their sophisticated paper, have well-documented the discovery of a new acute hypoxia sensor in CB type I cells, an olfactory receptor activated by lactate, Olfr78. The G-protein olfactory receptors are mainly expressed in olfactory epithelium where they activate signaling cascades leading to smell perception. However, the presence of an olfactory receptor in CB cells is not surprising; after their discovery in olfactory neurons, OL receptors were found in a variety of tissues with chemosensory functions (Kang and Koo, 2012). The involvement of Olfr78 in the CB oxygen sensing was evaluated comparing the respiratory response to hypoxia of *Olfr78*^−/−^ knockout mice with that of wild type animals (Chang et al., 2015). Defective animals had normal basal carotid sinus nerve discharge frequency but did not responded to hypoxic conditions (PaO_2_ = 60–80 mmHg). Importantly, in *Olfr78*^−/−^ mutants CB response to decreased pH was not affected demonstrating a specific deficiency in oxygen sensing and the presence of normal CB neurosecretory machinery in *Olfr78* null animals. The activation of Olfr78 by lactate was demonstrated *in vitro* by Chang et al. (2015). In HEK293T cells transfected with *Olfr78*, lactate activates Olfr78 with an EC_50_ of 4 mM. Importantly, lactate can be considered a physiological stimulus for CB since the EC_50_ is compatible with blood lactate concentration ranging between 1 and 5 mM. In addition, lactate failed to activate glomic cells in *Olfr78* defective animals. These findings strongly support the hypothesis that lactate activates glomic cells through Olfr78 signaling giving the molecular link to previous observations about the ability of lactate, even in the absence of hypoxia, to activate glomic cells, to stimulate breathing and to increase chemoafferent discharge. Moreover, the role of lactate in oxygen sensing in CB fits well with the discovery that cyanide, a mitochondrial poison that increases lactate concentration, elicits an important response by CB.

The paper by Chang et al. (2015) has uncovered a new important transduction mechanism of CB response to hypoxia. However, since olfactory receptors are able to couple to multiple G proteins activating adenylate cyclase (AC) or phospholipase-C (PLC) pathway (Ukhanova et al., 2014), the signaling downstream lactate-activated Olfr78 in CB needs to be further investigated. Indeed, it can differ from that of olfactory neurons: Olfr78 activation in CB could directly induce Ca^++^ transients or could lead to a K^+^ channel closure by an indirect mechanism. In this regard, NOX enzymes can be putative candidates. There are many examples of physiological responses mediated by NOX-activated membrane receptors (Damiano et al., 2012, 2015; Santillo et al., 2015), in some cases leading to the activation of ion channels (Lu et al., 2010). Previous studies have demonstrated that in *NOX2* knockout mice the response of glomic body to hypoxia is unaffected excluding the involvement of NADPH oxidase in oxygen sensing (He et al., 2002); however, since a characterization of NOX isoforms expression in glomic type I cells is completely lacking, it is not possible to exclude that other NOX isoforms acting in discrete membrane microdomains near to Olfr78 and redox-sensitive channels can be involved in the CB response to lactate.

The full hypoxic response of type I cells may require the concerted action of more than one mechanism, including lactate levels sensed by Olfr78. Multiple signaling pathways activated by hypoxic stimulus are integrated inside the cells leading to a fine modulation of neurotransmitter release and afferent fibers discharge according to the degree and timing of stimulation. In addition, the identification of lactate as physiologic ligand for Olfr78 opens the way for a new interpretation of previous data (Pluznick et al., 2013) demonstrating the presence of Olfr78, activated by short chain fatty acids (SCFAs), in the smooth muscle cells of the small resistance vessels and renal afferent arteriole. Olfr78 activation by gut microbiota-derived SCFA leading to an increase in renin release and vasoconstriction, was explained as finalized to counteract the hypotensive effects of SCFA mediated by their interaction with other receptors (Pluznick et al., 2013). Indeed, lactate-mediated Olfr78 activation in kidney and blood vessels could mediate an integrated ventilatory and cardiovascular response to hypoxia including, in addition to CB chemosensory reflex, a direct effect on vasculature and on the renin–angiotensin–aldosterone axis, resulting in an increase of perfusion pressure as adaptive responses to low O_2_ tension (Figure 1).

**Figure 1:**
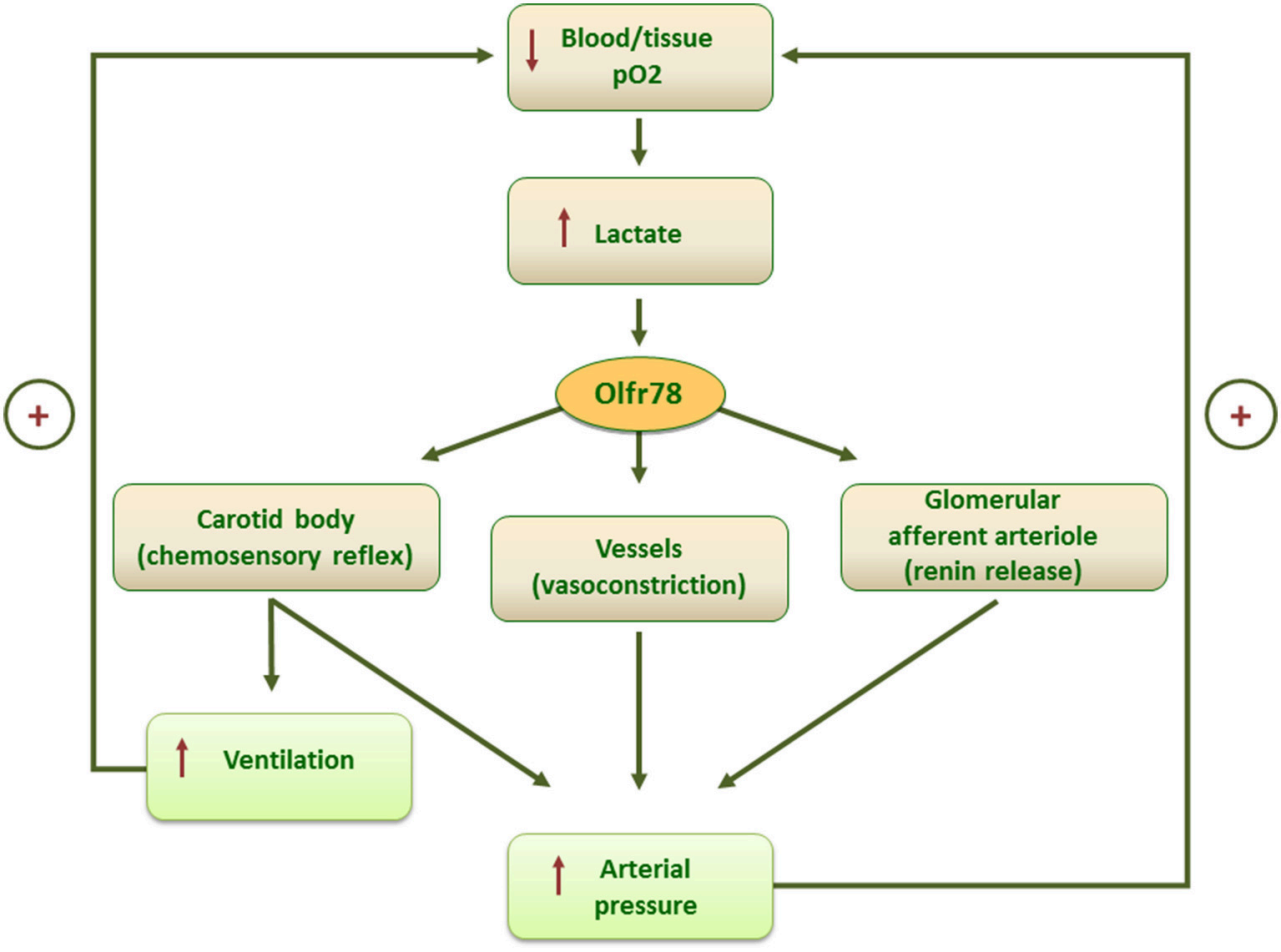
**Scheme of the putative mechanisms mediating the homeostatic control of blood/tissue pO_**2**_ by lactate-activated Olfrt8 signaling**.

## Author contributions

MS, conceived and wrote the article; SD, contributed to writing and graphics.

### Conflict of interest statement

The authors declare that the research was conducted in the absence of any commercial or financial relationships that could be construed as a potential conflict of interest.

## References

[B1] Chang A. J. Ortega F. E. Riegler J. Madison D. V. Krasnow M. A. (2015). Oxygen regulation of breathing through an olfactory receptor activated by lactate. Nature 527, 240–244. 10.1038/nature15721 26560302 PMC4765808

[B2] Damiano S. Fusco R. Morano A. De Mizio M. Paternò R. De Rosa A.. (2012). Reactive oxygen species regulate the levels of dual oxidase (DUOX1-2) in human neuroblastoma cells. PLoS ONE 7:e34405. 10.1371/journal.pone.0034405 22523549 PMC3327694

[B3] Damiano S. Morano A. Ucci V. Accetta R. Mondola P. Paternò R.. (2015). Dual oxidase 2 generated reactive oxygen species selectively mediate the induction of mucins by epidermal growth factor in enterocytes. Int. J. Biochem. Cell Biol. 60, 8–18. 10.1016/j.biocel.2014.12.014 25562511

[B4] He L. Chen J. Dinger B. Sanders K. Sundar K. Hoidal J.. (2002). Characteristics of carotid body chemosensitivity in NADPH oxidase-deficient mice. Am. J. Physiol. Cell Physiol. 282, C27–C33. 11742795 10.1152/ajpcell.2002.282.1.C27

[B5] Kang N. N. Koo J. H. (2012). Olfactory receptors in non-chemosensory tissues. BMB Rep. 45, 612–622. 10.5483/BMBRep.2012.45.11.232 23186999 PMC4133803

[B6] Kumar P. Prabhakar N. R. (2012). Peripheral chemoreceptors: function and plasticity of the carotid body. Comp. Physiol. 2, 141–219. 10.1002/cphy.c100069 23728973 PMC3919066

[B7] Lopez-Barneo J. Pardal R. Ortega-Sáenz P. (2001). Cellular mechanism of oxygen sensing. Annu. Rev. Physiol. 63, 259–287. 10.1146/annurev.physiol.63.1.259 11181957

[B8] Lu T. Zhang D. M. Wang X. L. He T. Wang R. X. Chai Q.. (2010). Regulation of coronary arterial BK channels by caveolae-mediated angiotensin II signaling in diabetes mellitus Circ. Res. 106, 1164–1173. 10.1161/CIRCRESAHA.109.209767 20167931 PMC2927991

[B9] Pluznick J. L. Protzko R. J. Gevorgyan H. Peterlin Z. Sipos A. Han J.. (2013). Olfactory receptor responding to gut microbiota-derived signals plays a role in renin secretion and blood pressure regulation. Proc. Natl. Acad. Sci. U.S.A. 110, 4410–4415. 10.1073/pnas.1215927110 23401498 PMC3600440

[B10] Santillo M. Colantuoni A. Mondola P. Guida B. Damiano S. (2015). NOX signaling in molecular cardiovascular mechanisms involved in the blood pressure homeostasis. Front. Physiol. 6:194. 10.3389/fphys.2015.00194 26217233 PMC4493385

[B11] Ukhanova K. Bobkova Y. Coreya E. A. Ache B. W. (2014). Ligand-selective activation of heterologously-expressed mammalian olfactory receptor Cell Calc. 56, 245–256. 10.1016/j.ceca.2014.07.012 25149566 PMC4188773

